# Changes in chemotherapy usage and outcome of early breast cancer patients in the last decade

**DOI:** 10.1007/s10549-016-4016-4

**Published:** 2016-10-15

**Authors:** A. Hennigs, F. Riedel, F. Marmé, P. Sinn, K. Lindel, A. Gondos, K. Smetanay, M. Golatta, C. Sohn, F. Schuetz, J. Heil, A. Schneeweiss

**Affiliations:** 1 Department of Gynecology and Obstetrics, University of Heidelberg, Heidelberg, Germany; 2 National Center for Tumor Diseases (NCT), University of Heidelberg, Heidelberg, Germany; 3 Department of Pathology, University of Heidelberg, Heidelberg, Germany; 4 Department of Radiation Oncology, University of Heidelberg, Heidelberg, Germany; 5 Division of Clinical Epidemiology and Aging Research, German Cancer Research Center (DKFZ), Heidelberg, Germany

**Keywords:** Early breast cancer, Pathologic complete response, Neoadjuvant chemotherapy, Adjuvant chemotherapy, Certified breast cancer unit

## Abstract

**Background:**

During the last decade, neoadjuvant chemotherapy (NACT) of early breast cancer (EBC) evolved from a therapy intended to enable operability to a standard treatment option aiming for increasing cure rates equivalent to adjuvant chemotherapy (ACT). In parallel, improvements in the quality control of breast cancer care have been established in specialized breast care units.

**Patients and methods:**

This study analyzed chemotherapy usage in patients with EBC treated at the Heidelberg University Breast Unit between January 2003 and December 2014.

**Results:**

Overall, 5703 patients were included in the analysis of whom 2222 (39 %) received chemotherapy, 817 (37 %) as NACT, and 1405 (63 %) as ACT. The chemotherapy usage declined from 48 % in 2003 to 34 % in 2014 of the cohort. Further, the proportion of NACT raised from 42 to 65 % irrespective of tumor subtype. In addition, frequency of pathologic complete response (pCR) defined as no tumor residues in breast and axilla (ypT0 ypN0) at surgery following NACT increased from 12 % in 2003 to 35 % in 2014. The greatest effect was observed in HER2+ breast cancer with an increase in patients achieving pCR from 24 to 68 %.

**Conclusions:**

The results mirror the refined indication for chemotherapy in EBC and its preferred usage as NACT in Germany. The increase in pCR rate over time suggests improvement in outcome accomplished by a multidisciplinary decision-making process and stringent measures for quality control.

**Electronic supplementary material:**

The online version of this article (doi:10.1007/s10549-016-4016-4) contains supplementary material, which is available to authorized users.

## Introduction

The insight of early breast cancer (EBC) as a systemic disease was one of the fundamental breakthroughs in breast cancer research and constitutes the basis for our therapy decision making today. As a consequence, adjuvant systemic therapy has become a backbone of EBC treatment. In particular, the evolution of chemotherapy has contributed substantially to the improved outcome of EBC patients today [1]. That includes not only the introduction of innovative cytotoxic drugs such as anthracyclines and taxanes but also the development of novel treatment approaches, i.e., dose-dense regimens and preoperative or neoadjuvant therapy.

Neoadjuvant chemotherapy (NACT) started as a treatment option to enable or improve operability of inflammatory, locally advanced or large breast cancer tumors. After equivalence in survival was confirmed, both adjuvant chemotherapy (ACT) and NACT became the standard treatment options for operable disease [2, 3]. NACT, however, offers a number of unique benefits over ACT. It improves the rate of breast-conserving surgery [2], allows an in vivo testing for drug sensitivity, and provides important prognostic information. The achievement of a pathologic complete response (pCR) defined as no invasive tumor residue in the breast and axilla following NACT is associated with improved disease-free and overall survival with the strongest correlation in aggressive breast cancer subtypes [4, 5]. At the patient level, this has been confirmed recently by a large meta-analysis [6]. Furthermore, post-neoadjuvant treatment may be differentiated depending on whether a patient achieves pCR after NACT or not. This is currently investigated in several trials.

A breast cancer patient’s prognosis also depends on the quality of treatment. To ensure patients are provided with a standard-of-care treatment, the German Cancer Society (DKG) and the German Society for Breast Diseases (DGS) introduced a certification system in 2003. It builds on the sustainable implementation of clinical guidelines in everyday clinical care, the establishment of multidisciplinary teams, and a quality assurance system [7]. The Heidelberg University Breast Care Unit (BCU) was fully certified by the certification board of DKG and DGS in 2003.

We analyzed chemotherapy usage in routine clinical care of a prospective cohort of 5703 patients with primary, non-metastatic breast cancer treated at a specialized BCU between January 2003 and December 2014.

## Patient and methods

### Patient selection

Data on medical history, demographic characteristics, diagnostics, therapy, and follow-up of all patients referred to the Heidelberg BCU for diagnosis and treatment of primary breast cancer have been prospectively documented since January 1, 2003 in our database. Patients were managed under certified conditions verified continuously by a re-certification process.

**Fig. 1:**
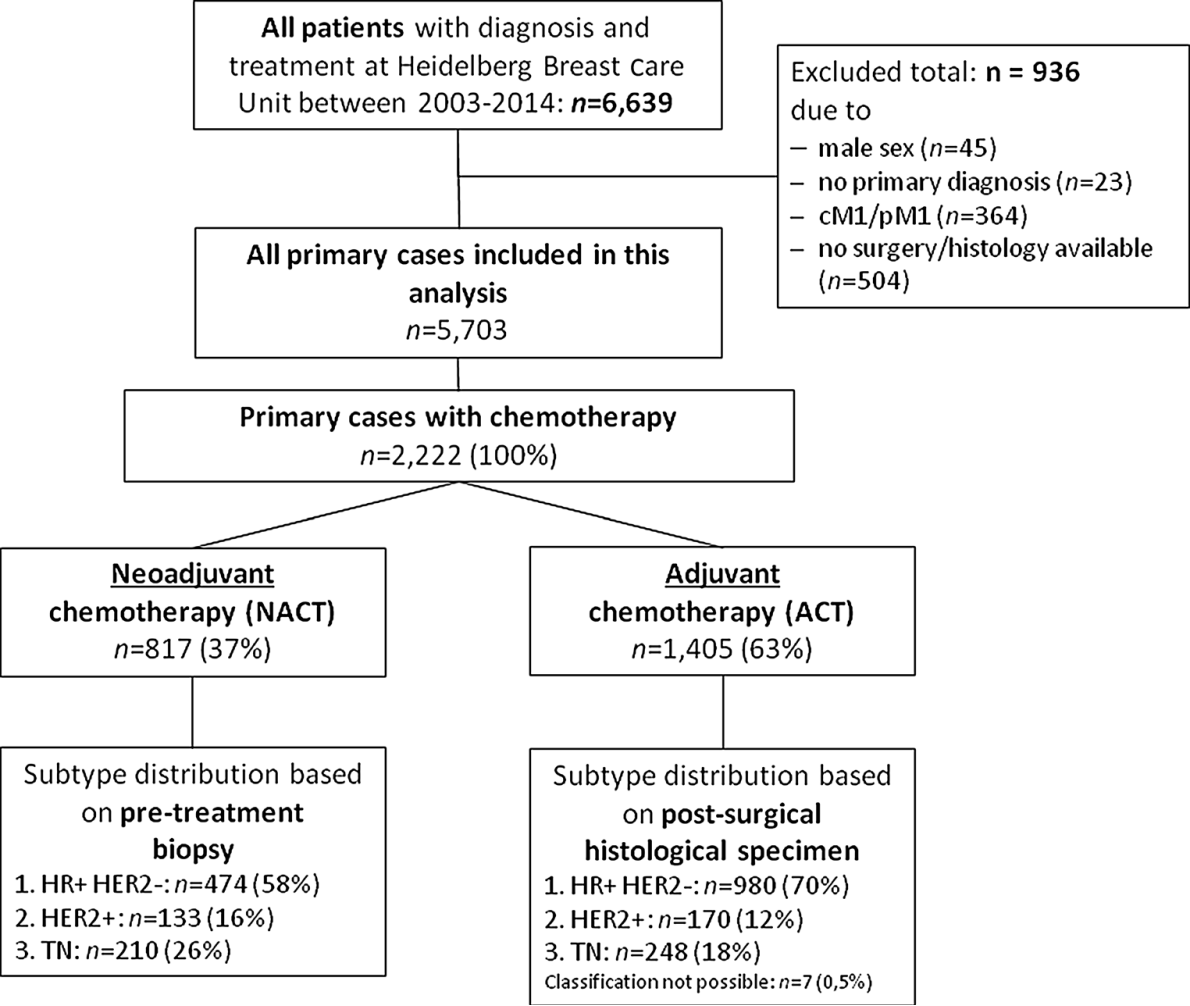
CONSORT diagram

Our prospective database comprised 6639 patients with primary breast cancer diagnosed and treated at Heidelberg BCU between January 1, 2003 and December 31, 2014. Nine hundred and thirty-six patients were excluded from the analysis due to male sex (*n* = 45), no primary diagnosis (*n* = 23), M1 status at time of diagnosis (*n* = 364), no surgery, or no histopathological report (*n* = 504). The selection process is outlined as a Consort diagram in Fig. 1. Patients had provided written informed consent on the use of their demographic and treatment data.

### Definitions of tumor histology and stages

Tumor histology was defined according to the World Health Organization criteria [8]; grading was performed and grouped into stages according to the most recent TNM classification [9].

The expression of estrogen receptor (ER), progesterone receptor (PgR), human epidermal growth factor receptor 2 (HER2), and Ki-67 was assessed using formalin-fixed paraffin-embedded tumor tissue according to international standards. Positivity for ER and PgR was defined as an immunoreactive score (IRS) of Remmele and Stegner of ≥1 out of 12 or as a total score (TS) of Allred of ≥1 out of 8. Moreover, any positive staining (i.e., ≥1 %) was defined as positive in accordance with the recent American Society of Clinical Oncology (ASCO)/College of American Pathologists (CAP) guideline recommendations [10]. HER2 status was defined as positive in case of a semiquantitative HercepTest© score of 3+ by immunohistochemistry (IHC) or in case of a positive FISH/CISH assay as per ASCO/CAP guideline recommendations [11].

Based on ER, PgR, and HER2-status by IHC ± FISH/CISH, three breast cancer subtypes were defined: 1) negative ER, PgR, and HER2 status corresponding to triple negative (TN); 2) positive HER2 status to HER2-positive (HER2+) irrespective of ER and PgR status; and 3) hormone receptor (HR)-positive status defined as a positive ER or a positive PgR status along with a negative HER2 status (HR+ HER2−). For patients with NACT, immunohistochemical information was based on the pre-treatment biopsy, for patients with ACT on the final post-surgery pathological sample.

### Statistical analysis

Data were analyzed descriptively using SPSS software version 22 (IBM, Armonk, USA). Annual and biannual percentages of chemotherapy use were calculated and presented as a longitudinal time trend analysis for the period from 2003 to 2014.

## Results

### Patient and tumor characteristics

The final cohort comprised 5703 patients of which 2222 patients received chemotherapy, 1405 (63 %) as ACT, and 817 (37 %) as NACT (Fig. 1). Demographic and tumor characteristics are presented separately for patients with ACT and NACT (Table 1). In the ACT cohort, the median age of patients was 54 years, more than half of the women were postmenopausal (54 %), and had a tumor grading of G3 in 41 % of the cases. Almost half of the patients had a tumor ≤2 cm (48 %) and were node-negative (48 %).

**Table 1:** Patient and tumor characteristics of all female cases with primary, non-metastatic breast cancer who were diagnosed and treated at Heidelberg University Breast Care Unit between 01.01.2003 and 31.12.2014 and underwent chemotherapy (*n* = 2222)

	Adjuvant chemotherapy (*n* = 1405)		Neoadjuvant chemotherapy (*n* = 817)	
	Number	%	Number	%
Age at diagnosis in years				
Median	54 years		49 years	
<51	576	41	469	57
51–65	566	40	267	33
>65	263	19	81	20
Menopausal status				
Pre	593	43	425	53
Peri	45	3	53	7
Post	740	54	319	40
Missing	27	–	20	–
Affected breast				
Left	704	50	420	51
Right	701	50	397	49
Main tumor histology				
Invasive carcinoma no specific type	1185	84	662	81
Invasive lobular carcinoma	180	13	122	15
Other (e.g., invasive medullar/mixed)	40	3	33	4
Post-surgical T-stage				
T0	0	0	223	29
T1	659	47	310	38
T2	569	41	153	19
T3	118	8	59	7
T4	22	2	22	3
Tis	0	0	44	5
Tx	37	3	7	1
Post-surgical N stage				
N0	674	48	540	66
N1	374	27	165	20
N2	179	13	67	8
N3	135	10	32	4
Nx	43	3	13	2
Grading				
Grade 1	66	5	15	2
Grade 2	751	55	307	45
Grade 3	559	41	361	53
Missing	29	–	134	–
Estrogen receptor				
Positive	1040	74	524	64
Negative	360	26	293	36
Missing	5	–	0	–
Progesterone receptor				
Positive	958	68	470	58
Negative	440	31	347	43
Missing	7	–	0	–
HER2 receptor				
Positive	193	14	133	16
Negative	1206	86	684	84
Missing	6	–	0	–
Ki-67				
≤14 %	421	36	139	22
>14 %	764	65	504	78
Missing	220	–	174	–

Patients in the NACT cohort tended to be younger (median age 49 vs. 54 years), had a higher proportion of G3 tumors (53 vs. 41 %), and had more often tumors with a Ki-67 >14 % (78 vs. 65 %) than patients who received ACT. As expected, the tumor size distribution following NACT shows a shift to smaller tumors with a relevant percentage of ypT0 stage (29 %) and lower proportions of (y)pT1 and (y)pT2 tumors (38 vs. 47 % and 19 vs. 41 %, respectively). In the NACT cohort, 58 % of patients had a HR+ HER2−, 16 % a HER2+ , and 26 % a TN subtype as compared to the ACT cohort with 69 % HR+ HER2−, 14 % HER2+, and 17 % TN.

### Chemotherapy usage

**Fig. 2:**
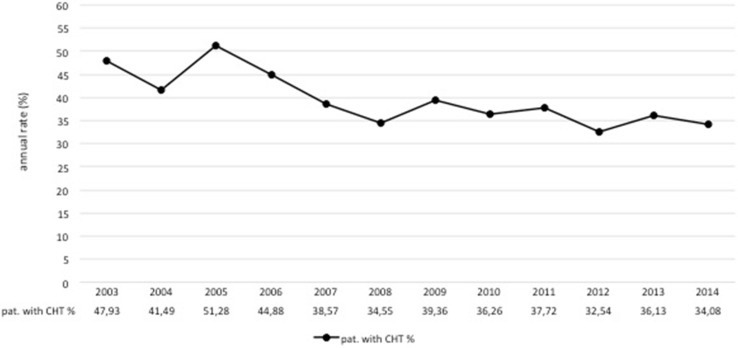
Overall proportion of patients receiving chemotherapy among primary, non-metastatic breast cancer patients at Heidelberg Breast Care Unit from 2003 to 2014

**Fig. 3:**
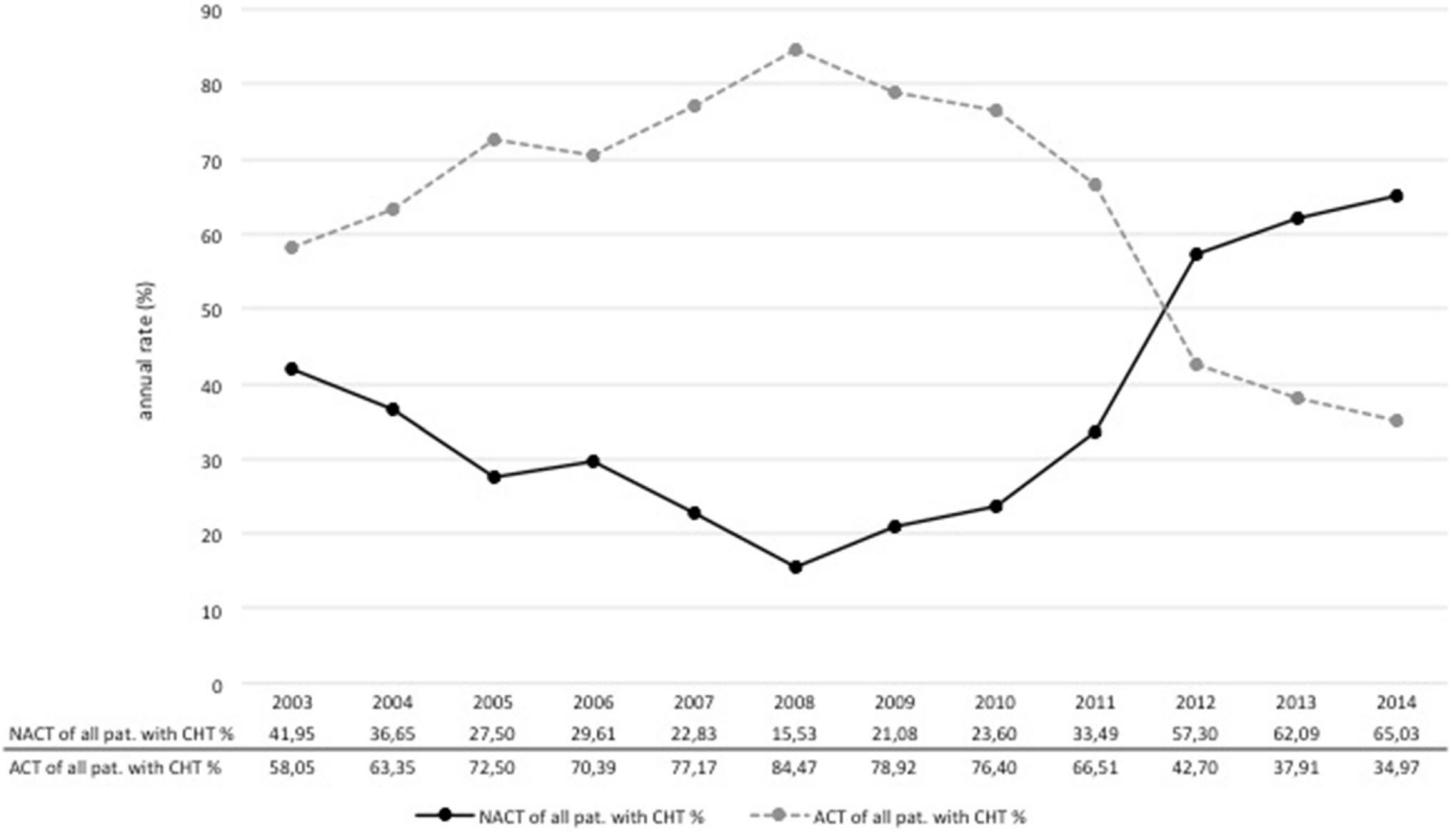
Relative proportion of adjuvant and neoadjuvant chemotherapy at Heidelberg Breast Care Unit from 2003 to 2014

**Fig. 4:**
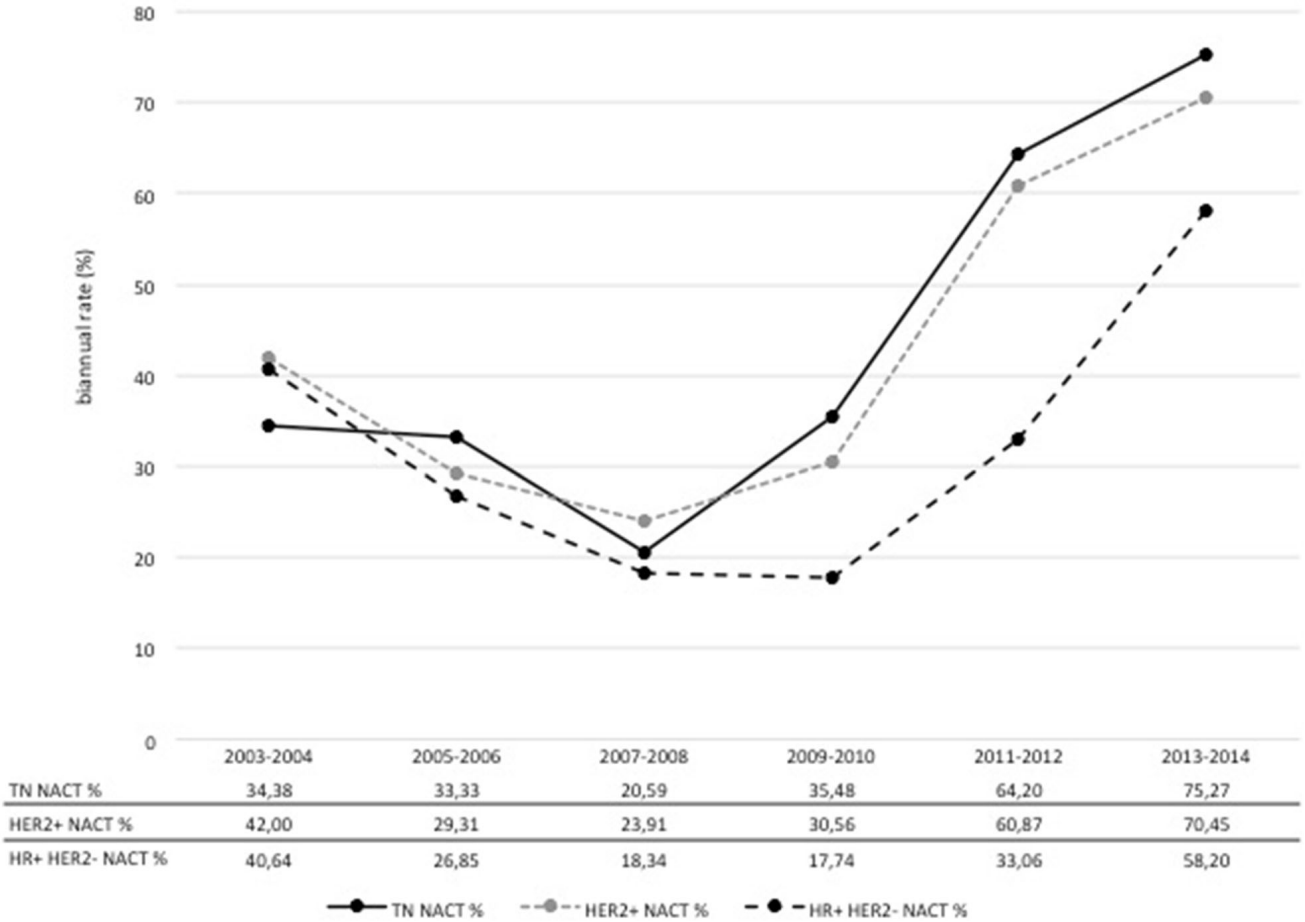
Relative proportion of NACT by breast cancer subtype (*n* = 2215, 7 missing) at Heidelberg Breast Care Unit from 2003 to 2014

Overall, the annual number of patients diagnosed and treated at our center increased over time, while chemotherapy usage declined. In 2003, 48 % of patients with EBC received chemotherapy compared to only 34 % in 2014 (Fig. 2). This is a steady decrease in the usage of chemotherapy from 2003 to 2014 of 14 %. In parallel, there was a shift in the relative proportions from ACT toward NACT. Figure 3 demonstrates a peak of 84 % for the use of ACT in 2008. The relative proportion of NACT increased continuously thereafter with a steep rise from 2011 to 2012. In 2014, the use of ACT dropped to 35 %, whereas the use of NACT amounted to 65 % (Fig. 3). The shift from ACT to NACT was seen irrespective of breast cancer subtype (Fig. 4). The relative proportion of NACT by breast cancer subtype in 2014 was highest in TN tumors with 75 %, following HER2+ tumors with 70 % and in HR+ HER2− tumors with 58 %.

### T-stage distribution

The pT-stage distribution of patients receiving ACT remained stable over the whole period under review (see Supplementary Fig. S1a). However, the relative proportion of ypT-stages in the NACT cohort changed considerably over time. Overall, there was an evolution to more favorable stages after admission of NACT. The percentage of patients with a postoperative ypT0 stage rose continuously from 16 % in 2003/2004 to 37 % in 2013/2014 and the rate of ypTis increased from 3 to 6 %, respectively. At the same time, the relative proportion of ypT1 stages decreased from 48 to 37 % and that of ypT3/4 stages from 16 to 5 %, respectively (see Supplementary Fig. S1b).

### Response to neoadjuvant chemotherapy

**Fig. 5:**
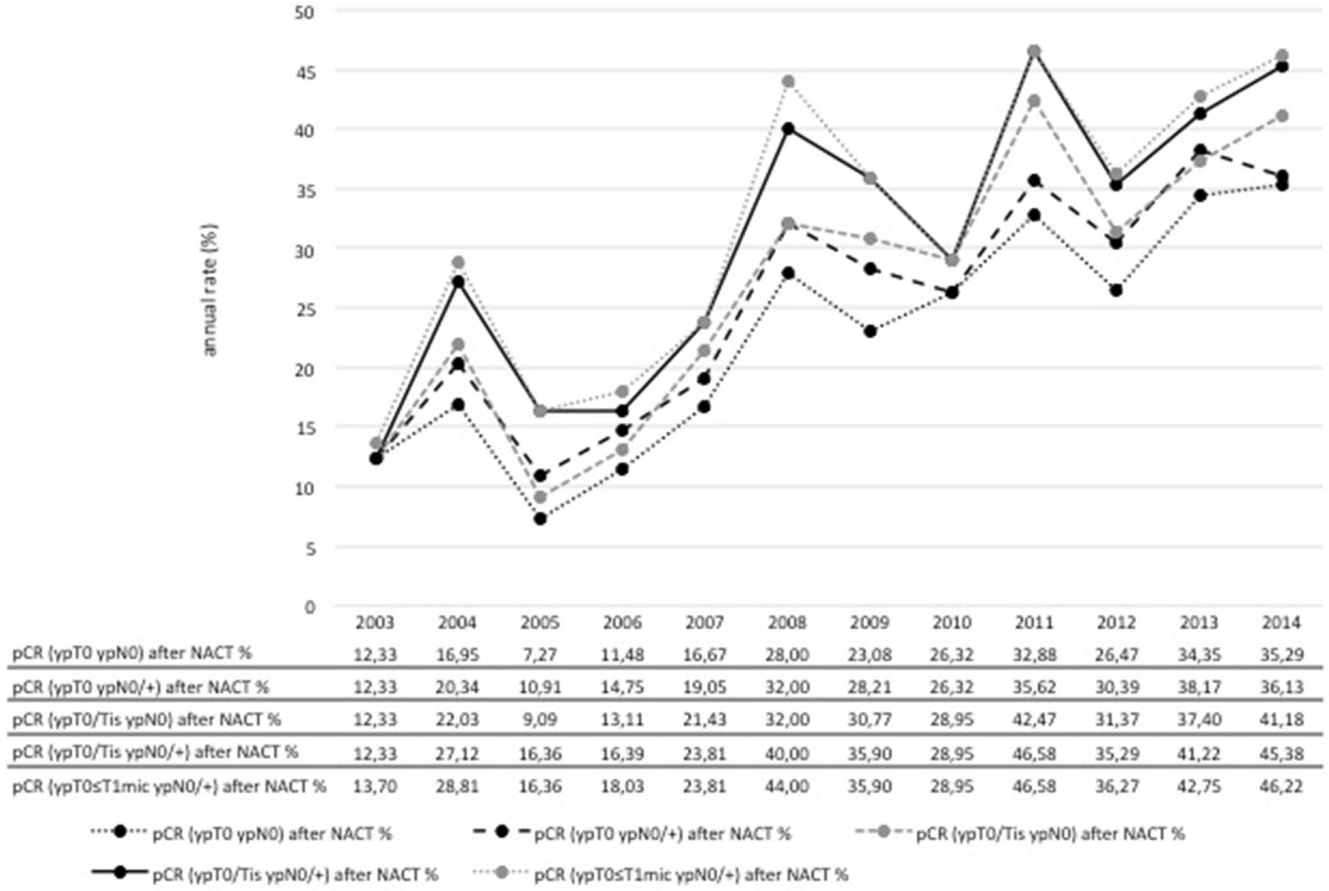
pCR according to different definitions of pCR at Heidelberg Breast Care Unit from 2003 to 2014

**Fig. 6:**
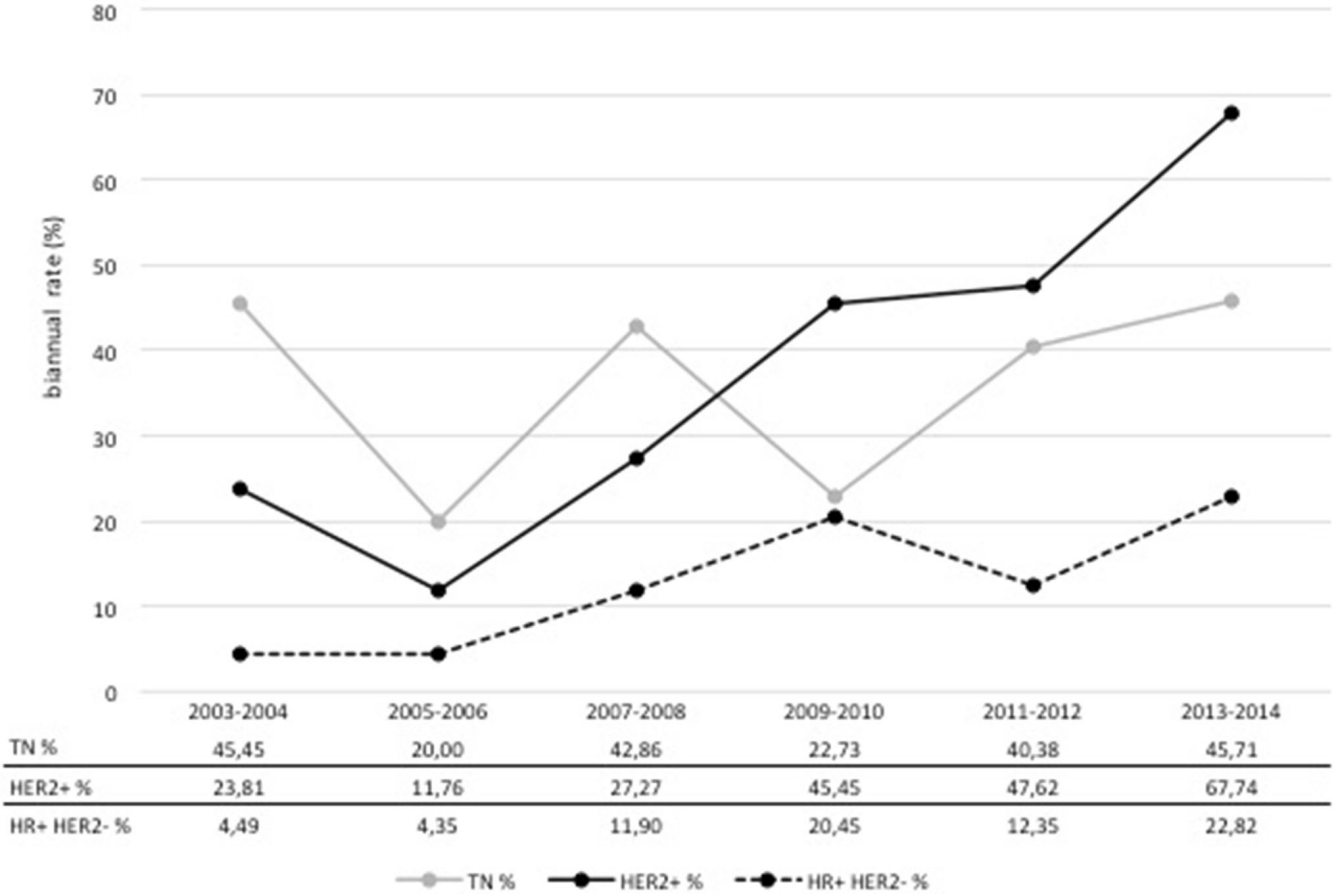
pCR (ypT0ypN0) by breast cancer subtype at Heidelberg Breast Care Unit from 2003 to 2014. The graph shows the pCR (ypT0ypN0) among patients receiving neoadjuvant chemotherapy in the three subgroups of immunohistochemically defined breast cancer subtypes at Heidelberg Breast Care Unit from 2003 to 2014

The shift to more favorable postoperative ypT-stages in the NACT cohort corresponded with an increase in the proportion of patients achieving a pCR over time (Fig. 5). PCR rate according to the most stringent definition (ypT0 ypN0) was 12 % in 2003 rising to 35 % in 2014. The effect was most prominent in tumors of the HER2+ subtype with pCR rising from 24 % in 2003/2004 to 68 % in 2013/2014. An increase in pCR was also seen in HR+ HER2− tumors with a rise from 4 to 23 %, respectively. However, there was no clear trend regarding pCR rates in TN disease (Fig. 6).

## Discussion

Our results reflect the fundamental developments in the usage of chemotherapy in early breast cancer patients. Since the advent of molecular classification systems, it has become evident that systemic therapy of EBC needs to be tailored according to individual risk factors and intrinsic subtype. Modern microarray-based gene expression profiles are the best way to visualize the heterogeneity of breast cancer with a more widely use in clinical routine in recent years. Patients that can be spared chemotherapy have been identified [2, 12]. Thus, it is reassuring that this leads to a substantial decline of overall chemotherapy use in EBC over time.

Another finding of our analysis was the considerable shift from ACT to NACT including a major increase of NACT from 2011 to 2012. To some extent, this may reflect the altered understanding and recognition of NACT. In 2009, the consensus conference of St. Gallen still stated that neoadjuvant systemic therapy was considered justified primarily to enhance the possibility of breast-conserving surgery [13]. However, in 2011, the prognostic value of NACT was finally acknowledged by the St. Gallen consensus conference [12]. NACT use in HER2+ tumors constitutes a significant proportion of the overall NACT use. Thus, the course of the curve may also have been influenced by the availability of trastuzumab outside clinical trials. The peak use of ACT and the minimum use of NACT were in 2007. In 2006, trastuzumab was first approved for adjuvant use in HER2+ EBC, and the application of trastuzumab in HER2+ disease as part of NACT was still deemed investigational [14]. Finally, however at the end of 2011, trastuzumab was also approved in combination with NACT.

While a survey in 2014 of a representative sample of 25 % of all German breast centers found a NACT rate of 15 % in all and of 26 % in highly specialized centers [15], 65 % of EBC patients at our center received NACT in the same period. This high rate is probably grounded on our longstanding engagement as an institution and as individual researchers in studying and developing NACT.

In addition, there was a clear improvement in the response to NACT. Our results compare favorably with the results of the major clinical trials. The pCR rate in all three subtypes documented in 2014 tended to be even higher than what is reported in the literature. While we reached pCR rates (ypT0 ypN0) of 46 % in TN, 68 % in HER2+ and 23 % in HR+ HER2− subtypes, the CTNeoBC pooled analysis reported rates of 34 % for TN, 31 % and 50 % for HER2+ HR+ and HER2+ HR− treated with trastuzumab, and 16 % for HR+ HER2− G3 based on a less conservative pCR definition (ypT0/is ypN0) [6]. The highest pCR (yp T0 ypN0) rates for HER2+ and for HER2+ HR– tumors reported so far were 52 and 84 % with chemotherapy plus trastuzumab and pertuzumab, respectively [16].

It is unlikely that this results from us using a different chemotherapy regimen or a major change in our choice of chemotherapy. In 2003, NACT was already anthracycline and taxane-based [17], and it still is in 2014 [2, 3]. One can argue that in HER2+ disease, the adoption of trastuzumab had an impact. Treatment of HER2+ patients with chemotherapy plus a dual HER2 blockade with trastuzumab and pertuzumab within clinical trials may have contributed to the further improvement from 2011/2012 to 2013/2014. But the improvement in outcomes probably also reflects our learning curve in selecting the appropriate patients with aggressive tumor biology for NACT. We believe they are also the result of the rigorous quality management and control of Heidelberg as a certified BCU. Moreover, it was shown that participation in clinical trials is associated with better outcomes [18].

Finally, achieving pCR is associated with a better prognosis for the individual patient, in particular, for those with aggressive breast cancer subtypes like HR+ G3, HER2+, and TN breast cancer [4–6]. Thus, it is reassuring that we improved in providing this clinical benefit to EBC patients.

## Electronic supplementary material

Below is the link to the electronic supplementary material.

Supplementary Figure 1 (TIFF 48 kb)

Supplementary Figure 2 (TIFF 52 kb)
