# Supplementary figures and images for: Changes in chemotherapy usage and outcome of early breast cancer patients in the last decade
Source: Breast Cancer Res Treat. 2016 Oct 15;160(3):491–9. doi: 10.1007/s10549-016-4016-4 (PMC5090013; doi:10.1007/s10549-016-4016-4)

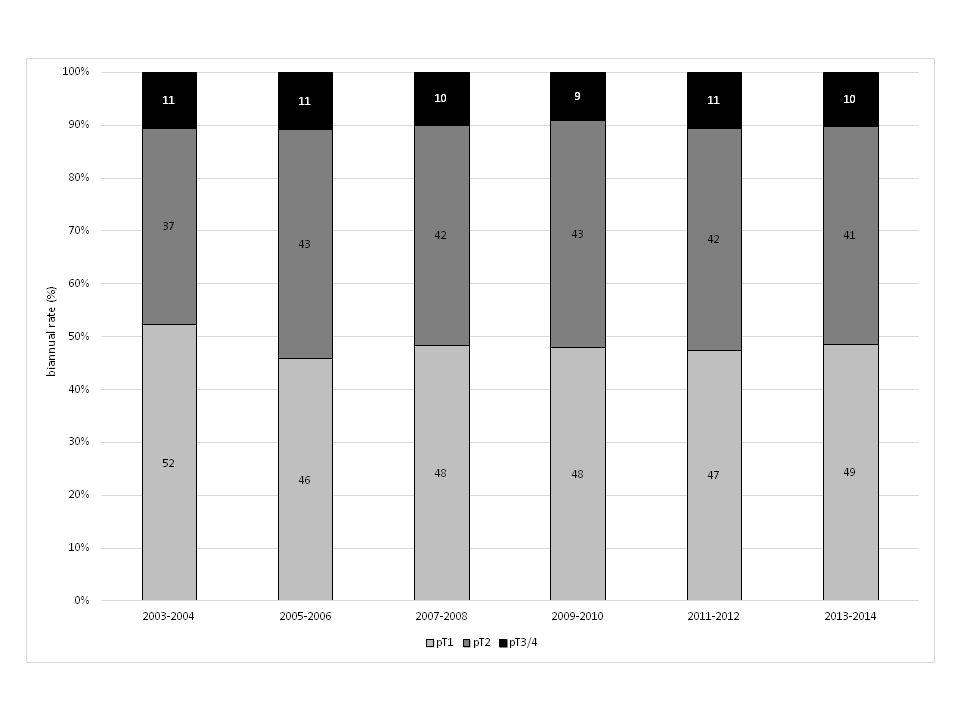

Supplement: Supplementary file 1 — Supplementary Figure 1 (TIFF 48 kb) [file 10549_2016_4016_MOESM1_ESM.tiff]

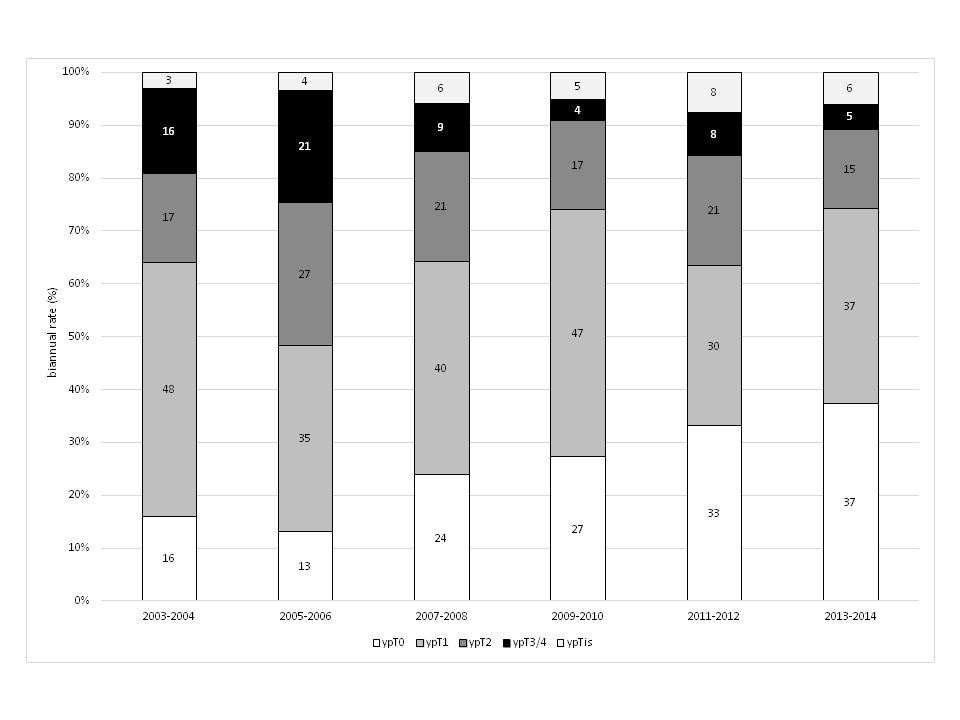

Supplement: Supplementary file 2 — Supplementary Figure 2 (TIFF 52 kb) [file 10549_2016_4016_MOESM2_ESM.tiff]
